# Poor maternal mental health is associated with a low degree of proactive control in refugee children

**DOI:** 10.1177/17470218231211573

**Published:** 2023-11-29

**Authors:** Gustaf Gredebäck, Marcus Lindskog, Jonathan Hall

**Affiliations:** Uppsala Universitet, Uppsala, Sweden

**Keywords:** Proactive control, prediction, child development, refugee, war, trauma

## Abstract

This study assesses the development of proactive control strategies in 100 Syrian refugee families (394 individuals) with 6- to 18-year-old children currently living in Turkish communities. The results demonstrate that children’s age and their mothers’ post-traumatic stress symptoms were associated with the degree of proactive control in their children, with worse mental health being associated with a larger reliance on reactive control and lesser reliance on proactive, future-oriented, control (measured via d′ in the AX-CPT task). None of the following factors contributed to children’s performance: fathers’ experience with post-traumatic stress, parents’ exposure to potentially traumatic war-related events, perceived discrimination, a decline in socio-economic status, religious beliefs, parents’ proactive control strategies, or the education or gender of the children themselves. The association between mothers’ mental health and proactive control strategies in children was large (in terms of effect size), suggesting that supporting mothers’ mental health might have clear effects on the development of their children.

The ability to act proactively, to organise one’s actions towards the future, is an essential ability that emerges during infancy (during action and social interaction; Gredebäck & Falck-Ytter, 2015; von Hofsten, 2004) and continues to develop during childhood (with the development of executive functions; Gottwald et al., 2016; Karr et al., 2018). As they get older, children gradually become more reliant on proactive control strategies (an early selection mechanism defined by anticipatory selection and maintenance of goal-related information; Braver, 2012; Gugelberg et al., 2021; see also Ossmy et al., 2022) and less reliant on reactive control (a late correction mechanism defined by stimulus- or event-driven activation of goal-relevant information; Fales et al., 2008; Filippi et al., 2022; Husa et al., 2021; Shields et al., 2016). A high degree of proactive control and low levels of reactive control (here referred to as the degree of proactive control) is predictive of school performance during childhood (Kubota et al., 2020) and work performance and labour market success during adulthood (Tornau & Frese, 2013).

At the same time, stress, anxiety, and traumatic experiences have the potential to work in the opposite direction, being associated with a lower degree of proactive control (Braver, 2012; Filippi et al., 2022). One reason for this experience-dependent shift towards reactivity might be functional. A high degree of proactive control might be most beneficial in stable environments where contextual cues reliably predict outcomes, while low levels of proactive control might be a better, more efficient, strategy in unreliable and unpredictable contexts (Lieder & Iwama, 2021). Another, potentially complementary, reason might be that a high degree of proactive control requires much more resources, and that stress and anxiety interfere with the ability to maintain information in working memory and hinder forward-oriented planning (Braver, 2012; Filippi et al., 2022; see also Yang et al., 2018). In fact, recent work demonstrates that visual working memory is negatively impacted by trauma experience in Syrian refugee children residing in Turkey (Mueller et al., 2021), with performance levels much lower than what would be expected from non-war exposed Western samples.

Given the importance of proactive control for life outcomes (as noted above), and for choices being made in the present, it is essential to investigate factors that promote a high degree of proactive control in families (parents and children) that live in contexts often associated with stress, anxiety, trauma, and unpredictable environments.

Despite the fact that children are heavily over-represented among the world’s refugees (1/3 of global population, 1/2 of all refugees; United Nations International Children’s Emergency Fund, 2022) and that one in six children in the world live in conflict zones (452 milion; Save the Children, 2021), surprisingly little psychological research has been devoted to this population. In this study, we ask how the degree of proactive control is expressed in children and parents experiencing war and trauma. More specifically, we focus on Syrian refugee families that live in Konya, Turkey. We take a family and intergenerational approach and aim to compare different transition models; that is, different ways in which children’s proactive/reactive control strategies can be affected by parental proactive/reactive strategies, parental mental health, and war-related experiences.

We contrast two theoretical models. *A direct transmission model* suggests that parents’ control strategies impact control strategies in their children directly, either through genetics or environment. More specifically, parents with a large degree of proactive control might create a context (in the broadest possible sense of the word, involving both genes, living conditions, and the interaction between the two) where such strategies are promoted and transferred to future generations. Findings consistent with this model have been reported for other cognitive capacities such as intelligence (Bradley & Corwyn, 2002) and executive functions in both traumatised (Chen et al., 2020) and typically developing children (Friedman et al., 2008; Sosic-Vasic et al., 2017). According to this model, high degrees of proactive control in parents should be associated with high degrees of proactive control in their children, and vice versa.

In contrast to this, a *parental wellbeing model* suggests that parents’ mental health impacts the quality of social interactions within the family. More specifically, that poor mental health and traumatic experiences create suboptimal raising practices and a different social climate in families that may have detrimental effects on the psychological development of children, including a lower degree of proactive control. Such effects have been observed with respect to both executive functions and social cognitive capacities in refugee children (Berg et al., 2019; Eltanamly et al., 2021; Gredebäck et al., 2021; Leen-Feldner et al., 2013; Michalek et al., 2022; Parfitt et al., 2014; Sack et al., 1995; Slone & Mann, 2016; van der Waerden et al., 2017; van Ee et al., 2012). Some of these effects have been primarily found in relation to maternal mental health in both Western, non-war-related contexts (Astor et al., 2020; Lundborg et al., 2021; Tu et al., 2021) and in Syrian children living in Turkey (Gredebäck et al., 2021). Young mothers are particularly vulnerable due to discrimination and experienced downward mobility (Peltonen et al., 2023). Other studies have connected child development directly to parents’ war-related experiences (Michalek et al., 2022) or to the quality of the father–child relationship (Scharpf et al., 2022). According to this model poor parental mental health and potentially traumatic war-related experiences should be associated with low degrees of proactive control in their children. Both models include factors in the environment that can impact child development, but the two models assume very different root causes.

In formulating these alternatives, it is important to note the possibility that both of these models operate at once (as is the case for executive functions, as noted in each example above). It is also possible that associations are not this straightforward and are more non-linear. For example, it has been demonstrated that more parental exposure to trauma sometimes is associated with better cognitive development in children (Qouta et al., 2021), possibly due to the presence of compensatory activities, such as more warmth, more focused, and positive parenting by adult household members that have experienced war and trauma (Eltanamly et al., 2021). So, these alternatives are not exclusive. Instead, they are included as model frameworks that allow us to assess the factors that shape the psychological development of children living in war and as refugees.

In this article, we will assess proactive control in refugee children and their parents and compare the two models of transition described above using the AX-CPT task (Braver, 2012; Chatham et al., 2009; Gonthier et al., 2019; Lucenet & Blaye, 2014). During this task, participants play a digital game in which they are asked to press different buttons based on a series of instructions. They should press one button in response to target cue “X” and another in response to cue “Y.” However, the button associated with “X” should only be pressed if the “X” is preceded by another cue “A,” that is, “A-X.” For all other combinations of cues (“B-X”; “A-Y”; “B-Y”), participants should press the button associated with “Y.” The “A-X” and “B-Y” combinations are most frequent (occurring 24 times each, other combinations occurring 6 times each; see Figure 1), creating pre-potent responses to press the button associated with “X” that can be proactively inhibited by paying attention to the preceding (contextual) cue “B.” One often used index that captures the degree of proactive control is d′ (z( Rate “A-X”) − *z*(False Alarms “B-X”)), *z* = z-transformed variables (for examples see Filippi et al., 2022; Gonthier et al., 2016; Kubota et al., 2020). Higher values indicate better proactive control and lower suggest that participants are more driven by reactive processes (that they incorrectly press the button associated with “X” when this cue appears, irrespective of prior contextual information; Braver, 2012; Chatham et al., 2009; Gonthier et al., 2019; Lucenet & Blaye, 2014), failing to use the predictive cue to regulate future behaviour. This combinatory dependent variable is well suited to compare the relationship between degree of proactive control and other variables in linear mixed models, as it combines hit rate and false alarms in a single variable, thereby reducing the number of statistical tests to one coherent analysis framework.

**Figure 1.**
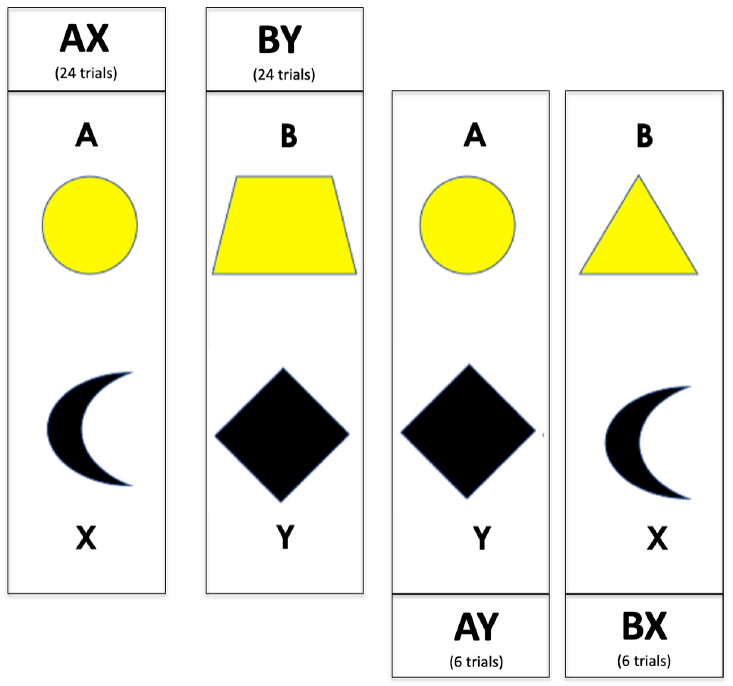
Stimuli used in the AX-CPT task. “A” and “X” stimuli are always the same, but “B” and “Y” categories are made up of five different geometric shapes, here exemplified with one item from each category.

A recent adult study (Gonthier et al., 2016) with six versions of the AX-CPT task reported an average error rate of approximately 6% (range 4.3–8.9) for AX and 11% for BX trials (range 4.4–20.3), resulting in a d′ of approximately 3 (range 2.44–3.39). In children, studies have documented that 3.5-year-olds appear to be dependent on reactive control strategies (Chatham et al., 2009) with an error rate of >50% and a d′ of 1.5. A general shift from reactive to proactive strategies in the AX-CPT task has been documented to occur between 5 and 6 years of age, developing in tandem with the development of working memory (Gonthier et al., 2019; Lucenet & Blaye, 2014 see also Troller-Renfree et al., 2020), with an error rate of 5%–15% and a d′ around 2.5 (Gonthier et al., 2019). Development continues with faster responses and more proactive control in both 9- and 12-year-old children and young adults (Lorsbach & Reimer, 2008, 2010). Twelve-year-olds have been reported to have an error rate of 10%–20% and a d′ of 2.2, whereas young adults had an error rate of 1%–4% and a d′ of >3.5 (Lorsbach & Reimer, 2008). The details of each experimental paradigm included in this section differs slightly, creating variance in error rates and d′ values across studies (Gonthier et al., 2016). It should also be noted that all of these studies are conducted in typically developing Western contexts and none focus on refugees, or families that have experienced war.

We analysed the association between performance on the AX-CPT task and a number of child and parent centred factors. In addition to age and gender of participants and their d′, we also included parents’ exposure to potentially traumatic events and their post-traumatic stress (PTS) as indicators of their war-related experiences and current mental health (Gredebäck et al., 2021; Michalek et al., 2022). In addition, we include a number of parent-centred variables that have been documented to impact refugee families and the development of children in this context, such as perceived discrimination, religiosity (as a sign of hope and/or an indication of social support), and downward mobility (Peltonen et al., 2023). The study also included education of children and parents and perceived chaos in the home, as we reasoned that these factors have the potential to impact the degree of proactive control in children.

## Method

### Participants

One hundred refugee families (174 adults [age: *M* = 39.8, *SD* = 7.8, range = [22, 60]; sex: 55.7% women] and 233 children [age: *M* = 12.2, *SD* = 3, range = [6, 18]; sex: 42.5% girls]) participated in the study (conducted between October 2019 and January 2020). The vast majority of children had mothers that were Arab (99%, 1% Turkmen), Sunni Muslims (99%, 1% Shia Muslims), from Syria (97%, 3% Iraq). Fathers were also mostly Arab (100%), Sunni Muslims (99%, 1% Shia Muslims), from Syria (98%, 2% Iraq). Syrian families were mostly from Aleppo (90%, remaining from Ar Raqqah, Deir al-Zour, Homes, Idlib, & Lattakia). Iraqi families came from Al Anbar, Babil, and Kirkuk.

Of these participants, 148 adults and 215 children completed the AX-CPT and were subsequently included in the current analysis. Families had left their homes during 2014–2016 (79%) and had, at the time of the study, been refugees for an average of 4.9 years (*SD* = 1.4 years). The number of children per family varied from 1 to 8 (median = 2 children). An opportunistic sample was used in the study, where participating families recommended the study to other families, as records of refugee families living in this community were not publicly available. The study was approved by the regional ethics review board in Sweden (2018-395) and the Necmettín Erbakan Universitesi in Turkey (2019/17). Each family received a monetary compensation equivalent of 10 Euro per participant for participation.

### Procedure and design

Each session started with tea and biscuits brought by the research assistants conducting the study (fluent in Arabic and Turkish). The study was described to the entire family (all family members participated at once, in a single session) and both written and verbal consent was obtained. Following this, each family member was seated in front of a laptop (DELL Vostro 3568, 15″ screen) with headphones using active noise reduction. All participating family members (children and adults) completed a list of experimental tasks and parents also filled out several questionnaires (see Table 1). Both the instructions and questions were in Arabic. The study lasted approximately 60 min for adults and 30 min for children, but there were large differences between families, as parents sometimes needed to help their children with instructions. Seating arrangements ensured that each adult had full privacy during testing (especially important when answering the questionnaires). Three papers have been published from this dataset focusing on social cognition and parents’ mental health (Gredebäck et al., 2021), maternal discipline and vulnerability among mothers (Peltonen et al., 2023), and intelligence in refugee children and their parents (Gredebäck et al., 2022). In sum, these papers demonstrate that children’s detection of emotional facial expressions is limited in children whose mothers suffer from poor mental health due to traumatic war experiences. A harsh parenting style among mothers, related to their own vulnerability, is associated with individual differences in this social cognitive ability. At the same time, neither children’s nor parents’ intelligence was associated with mental health or war experiences. Together, these findings suggest that the effects of war and trauma on children’s cognitive capacities are not uniform. So far, different transmission models have not been compared and children’s proactive control has not been included in any analysis beyond what is reported in this article.

**Table 1.** Experimental tasks (performed by all participants) and questions asked to parents, in the order listed.

Type	Focus area	Task	Reference
Experiment	Fluid intelligence	WASI matrix reasoning	(Wechsler, 1999)
Experiment	Attention	Visual search with emotional primes	(Haas et al., 2017)
Experiment	Social cognition	Emotional processing	(Gredebäck et al., 2021)^a^
Experiment	Proactive/reactive control	AX-CPT	(Gonthier et al., 2019)
Experiment	Risk-taking	BART	(van Ravenzwaaij et al., 2011)
Questionnaire	Demographics	Custom	(Gredebäck et al., 2021)
Questionnaire	Post-traumatic growth	PTGI-short form	(Cann et al., 2010)
Questionnaire	Home environment	CHAOS	(Matheny et al., 1995)^b^
Questionnaire	Home environment	HOME-SF	(Mott, 2004)^a^
Questionnaire	Psychosocial environment	FPSQ	(Garg & Dworkin, 2011)^a^
Questionnaire	Traumatic experiences	HTQ—part 1	(Mollica et al., 1992)
Questionnaire	Post-traumatic stress	PCL-C short form	(Lang & Stein, 2005)

a Minor adaptations to better fit the cultural context and test situation.

b Responses on a 4-point scale from very much like your home to not at all like your home, the original scale uses binary response options (yes/no).

In the AX-CPT task (Braver, 2012; Chatham et al., 2009; Gonthier et al., 2019; Lucenet & Blaye, 2014), participants are asked to respond to two sequential central target symbols on the screen. The symbols are mapped to two buttons (“left arrow” & “right arrow”). Within this paradigm there are two kinds of trials described as “special pair” and “normal pair” trials. The special “A-X” trials consist of two sequentially presented geometric shapes (“A”) and (“X”; see Figure 1), this is the most common pair of stimuli in the study. Participants are asked to press the “left arrow” button to the first stimulus (“A”) and the “right arrow” button to the second stimulus (“X”). On other pair trials, participants are asked to press the “left arrow” button for both the first and second stimuli (“A”-“Y,” “B”-“X,” or “B”-“Y”), for each set of stimuli (5 different geometric figures making up the category “X” and 5 other figures making up the category “Y,” each occurring on 10% of all trials). In many AX-CPT studies, the participants only press one button, to the second stimuli. In this case we added one more button press, to the first cue, to ensure that the participants paid attention to the stimuli, given that the study was conducted far from a standardised lab and in a more chaotic home environment. In total there are 60 trials, 24 “A”-“X” trials, 6 “A”-“Y” trials, 6 “B”-“X” trials, and 24 “B”-“Y.” All trials are presented in a pseudo-random order that ensured an approximately even distribution of events over the session. Prior to the actual AX-CPT task, children completed 15 practice trials to become accustomed to the response keys and instructions. Measures of participant response time (RT) and accuracy per trial were recorded. Each trial began with the presentation of a fixation point (“*”) centrally on the screen for 1,500 ms. A cue was then presented at the centre of the screen for 500 ms (“A” or “B”) followed by a second fixation marker (“*”) for 1,500 ms or 5,500 ms and the second stimulus (“X” or “Y”) for 500 ms. Following this sequence, participants had 3,000 ms to respond.

In the following analysis, d′ from the AX-CPT task was assessed in relation to a number of child and parent-centred factors (separately answered by mothers and fathers).^1^ Parents’ *potentially traumatic events* were assessed with the HTQ questionnaire, part 1 (Mollica et al., 1992), *post-traumatic stress* was assessed with the PCL-C short form (Lang & Stein, 2005), *perceived discrimination* was assessed with the question: “During the past 12 months have you ever been badly treated because of your foreign background? Please select all instances that apply.” Response options included six concrete situations (e.g., when looking for housing, during encounters in the street), an option to indicate other situations not listed, and the option to note that they had not been treated badly. The perceived discrimination variable was created by summing up the number of situations noted by participants (range 0–7). *Religiosity* was assessed with the question: “How strong are your family’s religious beliefs or practices? Response options ranged from extremely week to extremely strong in six steps (range 1–6). *Perceived chaos* in the home is measured with the CHAOS scale (Matheny et al., 1995). *Downward mobility* is calculated by subtracting the respondent’s perceived socioeconomic status (SES) at their point of origin from their current perceived SES in Turkey. The questions asked were:

Imagine the society in your country of origin (Syria or Iraq) as arranged on a scale like the one shown below, where the worst off socially and economically are on the left (0) and the best off are on the right (10). Please move the slider to select the place where you feel you stood prior to the war.

and

Imagine Turkish society as arranged on a scale like the one shown below, where the worst off socially and economically are on the left (0) and the best off are on the right (10). Please move the slider to select the place where you feel you stand.

*Education* of parents were assessed on a 6-point scale ranging from no formal education (1) to >12 years of schooling. *Education* of children was reported in terms of years of schooling (from 0 to 12). Only the mother’s response was used for this variable.

### Statistical analysis

Data from the AX-CPT task were preprocessed by removing all trials with an RT of <110 ms, based on Woods et al. (2015), excluding 258 trials from children and 127 trials from parents. The variable of interest d′ was calculated by computing a d′ index from hits on AX trials and false alarms on BX trials as *Z*(H)—*Z*(F), with H representing hits on AX trials, F representing false alarms on BX trials, and Z representing the z-transform of a value (see Table 2 for descriptive information from the task and Supplemental Material for the code used to calculate d′, based on Gonthier et al. (2016)).

**Table 2.** Descriptive data from the AX-CPT task.

	AX	BY	AY	BX
Trials presented (*n*)	24/24/24	24/24/24	6/6/6	6/6/6
Trials included (*n*)	17/20/19	20/22/22	4/5/5	3/3/3
Error rate (%)	28/14/21	15/9/7	26/15/15	54/50/57
Mean RT (ms)	760/781/775	761/757/766	838/842/855	832/874/848
d′				.55/1.14/.69

RT: response time.

Each cell includes data for children/mothers/fathers.

**Figure 2.**
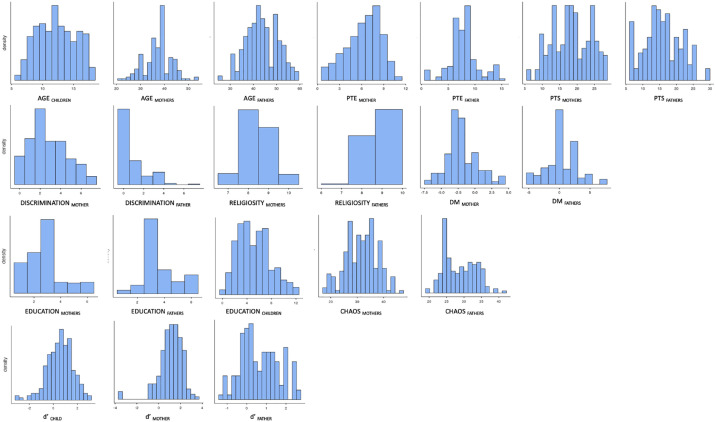
Histograms for each of the variables used in linear models below. *Note*: PTE: potentially traumatic events; PTS: post-traumatic stress; DM: downward mobility; d′: degree of proactive control.

A first set of analyses were performed to assess performance on the AX-CPT task, focusing on the degree of proactive control strategies used, as indicated by d′. Single sample *t*-tests against zero were performed separately for children, mothers, and fathers. This was followed by a linear mixed model that assessed how the degree of proactive control (d′) varied across these groups (specified as d′ ~ 1 + group(child, mother, or father)^2^ + (1| family)), performed in Jamovi (version 2.3.21.0) using the GAMLj module (version 2.6.6). Family ID intercept was set as a random factor, and restricted maximum likelihood (REML) parameter estimations were used. Post hoc analysis report Bonferroni corrected *p*-values. This approach was taken for all subsequent mixed models. All variables used in the analyses of this article are depicted as histograms in Figure 2. All these variables were within acceptable ranges for skewness (range −1.38, +1.96) and kurtosis (range −0.99, +4.72). The study only reports main effects, as no significant second-order interaction effects were observed in the analysis. Guide for interpreting: η^2^ = 0.01 indicates a small effect, η^2^ = 0.06 indicates a medium effect, and η^2^ = 0.14 indicates a large effect.

In a second step, a series of linear mixed models were performed to assess the relation between children’s d′ and specific family indicators. Model C1 assessed children’s d′ in relation to age and education level of the child and their parents (specified as: d′_child_ ~ 1 + age_child_ + education_child_ + education_mother/father_^3^ + (1| family)). Models C2_mother_ and C2_father_ included a broader range of family indicators along with any significant variables from model C1 (specified as: d′_child_ ~ 1 + age_child_ + gender_child_ + potentially traumatic events_mother OR father_ + post-traumatic stress_mother OR father_ + perceived discrimination_mother OR father_ + religiosity_mother OR father_ + perceived downward mobility_mother OR father_ + perceived chaos in the home_mother OR father_ + d′_mother OR father_ +(1| family)). Model C3 combined significant effects from models C1 and C2_mother/father_ (specified as: d′_child_ ~ 1 + Age_child_ + post-traumatic stress_mother/father_ + perceived chaos in the home_mother/father_ + (1| family).

Many of the variables included are based on either a small set of questions (perceived downward mobility or religiosity) or lists of different events that parents might have experienced (such as discrimination), and in these cases Cronbach’s alpha might not be appropriate. However, PTS, potentially traumatic events, and perceived chaos in the home fulfil criteria for assessing reliability. Cronbach’s alpha is good for both PTS of mothers (Cronbach’s alpha = .776) and fathers (Cronbach’s alpha = .771) as well as for perceived chaos in the home reported by mothers (Cronbach’s alpha = .734) and fathers (Cronbach’s alpha = .506). In the latter case, one question (nr 1) correlated negatively with the rest (−.353 for mothers, −.426 for fathers); this question was removed prior to this and subsequent analysis. Cronbach’s alpha for potentially traumatic events vary to a larger degree between parents with lower reliability for mothers (Cronbach’s alpha = .662) than fathers (Cronbach’s alpha = .901).

## Results

### Degree of proactive control

**Figure 3.**
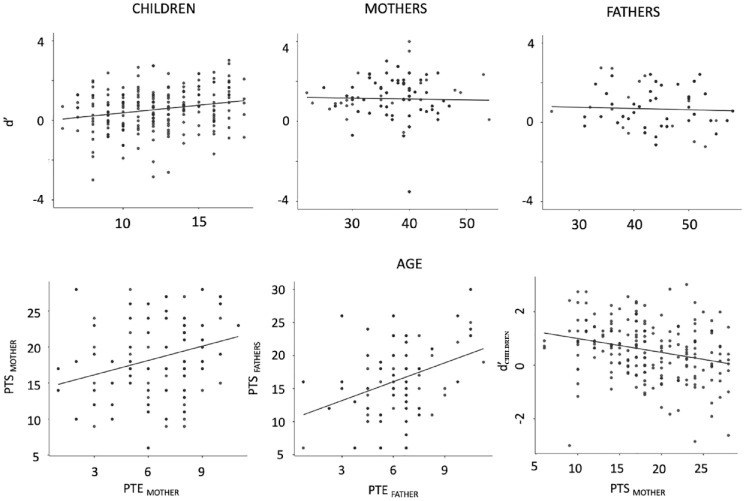
Upper: Scatterplots depicting degree of proactive control (d′) for individual participants and age (years), separate for children, mothers, and fathers. Horizontal lines mark a d′ of zero. Lower: Scatterplots depicting the association between potentially traumatic events (PTE) and post-traumatic stress (PTS), separate for mothers and fathers and the association between mothers’ PTS and children’s d′. Diagonal line depicts linear regression lines.

Descriptive data for d′ and RTs across the three age groups (children, mothers, fathers) are depicted in Table 2 and Figure 3 (for a complete correlation table see Supplemental Table 1). Single sample *t*-tests demonstrate that children, *t*(214) = 7.61, *p* < .001, Cohen’s *d* = .519, mothers, *t*(177) = 14.27, *p* < .001, Cohen’s *d* = 1.07, and fathers, *t*(137) = 8.37, *p* < .001, Cohen’s *d* = .712, significantly differs from, and has values higher than zero. A mixed model with d′ as dependent variable and group (child, mother, father) as a factor demonstrated a significant effect of group, *F*(1, 2) = 19, *p* < .001. Post hoc tests demonstrated that mothers performed better than children, *t*(474) = 6.14, *p* < .001, and fathers, *t*(497) = 3.39, *p* < .002. However, no difference was observed between fathers and their children, *t*(474) = 2.0, *p* = .14.

### Factors impacting degree of proactive control in children

The model C1 demonstrated a significant contribution of children’s age, *F*(1, 82.1) = 5.74, *p* = .019, η^2^_partial_ = .065; model fit: AIC = 431.27, BIC = 471.32, *R*^2^_marginal_ = 0.06, *R*^2^_conditional_ = 0.42, on children’s d′ with older age being associated with a higher d′; see Table 3 and Figure 3 for details regarding all child-centred models. Models C2_mother_ demonstrated that children’s age, *F*(1, 161.8) = 10.00, *p* = .002, η^2^_partial_ = .06, and mother’s PTS, *F*(1, 61.3) = 7.89, *p* = .007, η^2^_partial_ = .11, contributed to children’s d′ (model fit: AIC = 530.69, BIC = 611.25, *R*^2^_marginal_ = 0.14, *R*^2^_conditional_ = 0.31). Model C2_father_ demonstrated a significant contribution of children’s age, *F*(1, 121.5) = 6.09, *p* = .01, η^2^_partial_ = .05, and father’s perception of chaos in the home, *F*(1, 44.6) = 4.53, *p* = .04, η^2^_partial_ = .092, to children’s d′ (model fit: AIC = 407.51, BIC = 481.16, *R*^2^_marginal_ = 0.18, *R*^2^_conditional_ = 0.40). The reduced model C3 demonstrated that children’s age, *F*(1, 145.6) = 8.33, *p* = .004, η^2^_partial_ = .054, and maternal PTS, *F*(1, 54.35) = 13.00, *p* < .001, η^2^_partial_ = .19, are the only factors that explain children’s d′ (model fit: AIC = 4451.430, BIC = 509.30, *R*^2^_marginal_ = 0.19, *R*^2^_conditional_ = 0.39) when controlling for father’s PTS and both mother’s and father’s perceived chaos in the home.^4^

**Table 3.** Depict the results of models C1 assessing the association between children’s d′ and their own, and their parents, age, and education and C2_mother/father_ assessing the association between children’s d′ and parental (separate for mothers and fathers) characteristics, experiences, and current situation.

Model	Variable	Estimate	*SE*	CI_95_	*df*	*t*	*p*	η^2^_partial_
C1	***Age_C_***	0.12	0.05	0.02, 0.22	82.1	2.40	.02	.065
	Education_C_	−0.08	0.07	−0.21, 0.06	86.3	−1.16	.25	.02
	Education_M_	0.17	0.15	−0.12, 0.47	30.4	1.19	.24	.04
	Education_F_	0.02	0.15	−0.27, 0.31	37.8	0.16	.87	<.001
C2_mother_	(Intercept)	−0.09	0.91	−1.90, 1.73	76.7	−0.09	.92	<.001
	***Age_C_***	0.08	0.03	0.03, 0.13	161.8	3.16	.002	.06
	Gender_C_	0.026	0.16	−0.29, 0.34	163.5	0.16	.872	<.001
	PTE_M_	−0.03	0.04	−0.11, 0.06	64.6	−0.62	.537	.006
	***PTS_M_***	−0.05	0.02	−0.09, −0.02	61.3	−2.81	.007	.11
	Discrimination_M_	0.03	0.05	−0.07, 0.14	73.7	0.65	.516	.006
	Religiosity_M_	0.05	0.13	−0.20, −0.30	63.0	0.41	.682	.002
	DM_M_	−0.05	0.04	−0.14, 0.03	54.9	−1.25	.218	.028
	CHAOS_M_	0.01	0.02	−0.02, 0.04	71.6	0.47	.643	.003
	d′_M_	0.10	0.09	−0.08, 0.28	55.8	1.11	.273	.022
C2_father_	(Intercept)	−0.18	1.19	−2.57, 2.20	47.0	−0.15	.88	<.001
	***Age_C_***	0.07	0.03	0.01, 0.12	121.5	2.47	.015	.048
	Gender_C_	0.30	0.18	−0.04, 0.65	119.3	1.74	.085	.024
	PTE_F_	0.07	0.05	−0.02, 0.16	42.6	1.58	.122	.055
	PTS_F_	−0.01	0.02	−0.05, 0.03	36.3	−0.48	.635	.006
	Discrimination_F_	−0.06	0.09	−0.24, 0.12	57.1	−0.66	.513	.008
	Religiosity_F_	0.20	0.15	−0.11, 0.51	47.1	1.30	.200	.034
	DM_F_	0.01	0.04	−0.08, 0.10	37.7	0.33	.739	.002
	***CHAOS_F_***	−0.06	0.03	−0.11, −0.03	44.6	−2.13	.039	.092
	d′_F_	0.05	0.11	−0.18, 0.27	45.5	0.43	.669	.004
C3	(Intercept)	1.01	0.73	−0.40, 2.53	68.2	1.44	.15	.03
	***Age_C_***	0.07	0.02	0.02, 0.12	145.6	2.89	.004	.054
	***PTS_M_***	−0.07	0.02	−0.11, −0.03	54.3	−3.61	< .001	.19
	PTS_F_	0.007	0.02	−0.03, 0.04	47.7	0.39	.70	.003
	CHAOS_M_	0.02	0.02	−0.02, 0.06	56.3	0.92	.36	.015
	CHAOS_F_	−0.03	0.02	−0.08, 0.01	65.0	−1.41	.16	.029

CI: confidence interval; C: child, M: mother, F: father, PTE: potentially traumatic events, PTS: post-traumatic symptoms, DM: downward mobility; d′: degree of proactive control.

## Discussion

The aim of the current study was to assess if, and how, Syrian refugee children’s proactive control strategies are impacted by family experiences and their parents’ mental health. More specifically, we contrasted two transmission models, a direct transmission model (where parents’ control strategies impact the same strategies in their children) and a parental wellbeing model (where parents’ traumatic experiences and mental health impact control strategies in their children). The study demonstrates that Syrian refugee children’s proactive control strategies, or lack thereof, were impacted by maternal mental health, more specifically, the PTS symptoms of mothers, in statistical terms a large effect. Parents’ own proactive control strategies did not impact children’s proactive control. Together, these results speak against the direct transmission model and instead support the parental wellbeing model that emphasises the social and emotional climate among parents, and the mental health of mothers in particular, as important for child development.

To further elaborate on the parental wellbeing model in the current context, we here propose that maternal scaffolding and the overall psychosocial climate in the family is negatively affected when mothers suffer from poor mental health (in this case PTS). This includes a risk for a reduction in high-quality social interactions necessary for children (Astor et al., 2020), for inappropriate parental strategies (Peltonen et al., 2023), poor attachment quality (van Ee et al., 2016), and a general low level of expressed empathy (Salo et al., 2020), and support (Jacob & Johnson, 1997). This, less than optimal, environment has the potential to substantially alter the expectations children have about the world. In addition, increased stress and uncertainty tax working memory and lower the capacity to think ahead and plan future actions appropriately (Braver, 2012; Filippi et al., 2022; see also Yang et al., 2018). Together, these risk factors might lead to a stronger focus on reactive control strategies that may be functional for children living in these contexts, but unfortunately have potential long-term negative consequences for life outcomes, including school grades (Kubota et al., 2020), labour market success (Tornau & Frese, 2013), and general life satisfaction (Siebert et al., 2020).

Given the fact that these families have lost much of their material and social capital, these processes risk holding future generations back, cementing, or even increasing inequalities over generations. The economic literature discusses the risk of poverty traps, where families that are too poor to thrive will not benefit from modernisation, urbanisation, or other societal resources due to inadequate financial resources needed to invest in their own future (Banerjee & Duflo, 2012; Kraay & McKenzie, 2014). In the current context, it is perhaps possible to talk about a complementary mental health trap where mothers that have experienced hardship, and from these experiences suffer from poor mental health, are unable to provide the environment needed for their children to flourish—a situation that might be difficult to get out of on one’s own. Here, community support and an active involvement of non-governmental organisations (NGOs) and states are needed to support these mothers, and through them foster an environment that promotes proactive control strategies and a solid foundation for development across generations.

Before concluding, some important facets of the results require additional reflections. First, no interaction effects were observed, suggesting that the effects observed are consistent across ages, from the youngest 6-year-olds to the oldest 18-year-olds in the study, and similar for boys and girls. This is important to keep in mind when discussing models of vulnerability and plasticity from psychology (e.g., Nelson & Gabard-Durnam, 2020) and economics (e.g., Heckman et al., 2013) that often highlight that the largest impacts of interventions and exposure to adversity occur at the youngest ages. Vulnerability appears to continue throughout the protracted period of childhood and impact older children that spent their first years of life in relative prosperity in a pre-war Syrian context.

Second, it is interesting to note that the role of fathers is rather small, and non-existent in the final analysis controlling for maternal factors. At the same time the effect of mother’s mental health is large (as indicated by the effects sizes expressed in Model C3, Table 3). Similar lack of effects from fathers have been reported in the past (Gredebäck et al., 2021) and can perhaps be attributed to the financial demands that require many fathers to work away from home and to gender roles that emphasise maternal responsibility and care of children at home (El-Khani et al., 2016; Yaylaci, 2018).

Third, a noteworthy and important point to reflect on is the low d′ scores and high error rates reported in the current study when comparing with prior work in Western, non-trauma related, contexts. Prior work with adults reports error rates ranging from 6%–11% and d′ values around 3.5 (Gonthier et al., 2016), whereas prior work with 9- to 12-year-old children report error rates of 10%–20% and d′ values of around 2.5 (Lorsbach & Reimer, 2008, 2010). In the current study adults’ error rates ranged from 6% to 57% with d′ values averaging 0.69 for fathers and 1.14 for mothers. At the same time, children’s error rates ranged from 15% to 54%, with d′ values averaging .55, and the performance of fathers was not statistically significantly different from that of their children. Even when accounting for the fact that the age range is much wider in this study compared with other studies, it remains difficult to directly compare these results with those of prior studies just based on the vast difference in performance. As indicated by training sessions and the descriptive statistics in Table 2, the participants were able to perform the task. It is likely that the lower performance is due to the fact that the sample consists in its entirety of people that have suffered much hardship and are emotionally and psychologically taxed already at the start of the study. It may be that the group as a whole suffers from traumatic experiences and poor mental health in ways that tax working memory, making the task more difficult for this group compared with the healthy, non-traumatised, college students that usually take part in experimental studies in Western contexts.

It is possible that the addition of a second button press (to both the first and second cues) taxed the participants’ working memory and that this is a reason why participants perform worse than what has been reported in prior Western contexts. At the same time, similar findings, with lower performance levels in Syrian refugee children living in Turkey, have previously been reported for visual working memory (Mueller et al., 2021), strengthening the notion that this finding is not an artefact of the current experimental task, but instead a sign of the challenges that this group of children face due to their prior experiences and current living conditions. Another, possibly complementary, alternative is that the abstract nature of the stimuli was particularly challenging for the children that took part in this study, and that more ecologically relevant stimuli might have increased performance in this group (as previoulsy demonstrated with US children exposed to violence and poverty: Young et al., 2022).

Fourth, one factor that we did not assess was children’s own mental health and experience of potentially traumatic events, a factor that we know is highly impacted by war and experiences as a refugee (Frounfelker et al., 2020). This was a deliberate choice as we did not feel that we had the capacity to provide adequate support in the field to children if such questions triggered negative thoughts among participants. At the same time, it is perhaps difficult to completely separate mental health of parents (especially mothers in this context), their war-related experiences, and the mental health and resilience of their children. Future work will have to dive into this question and assess how maternal mental health, maternal war-related experience, and children’s own mental health interact with the psychological development of children. What we can say from the current results is that the larger psychosocial context of the child within the family impacts child development and that evidence could be found in support of the parental wellbeing transmission model.

What we need to do in the future is support mothers, and their children, to improve mental health, resilience, child development, and long-term outcomes for Syrian refugees living in neighbouring countries. It is quite possible that this effect is not isolated to this group of refugees and that similar results can be observed across conflicts and continents, including the ongoing war in Ukraine. At the same time, it has been demonstrated that the impact of maternal mental health on child development is not uniform across the globe (Astor et al., 2022) with unique culture specific risk and protective factors. As is often the case, more data are needed from other contexts, conflicts, and continents to assess the universality (or lack thereof) of these findings.

In summary, we demonstrate that maternal mental health is strongly associated with children’s ability to use the current context to predict future events, a psychological construct known as proactive control. Syrian refugee families where mothers have suffered much hardship and experience poor mental health (as measured by PTS symptoms) have children that do worse on this task than mothers from the same context that suffer less from poor mental health.

## Supplemental Material

sj-docx-1-qjp-10.1177_17470218231211573 – Supplemental material for Poor maternal mental health is associated with a low degree of proactive control in refugee children Supplemental material, sj-docx-1-qjp-10.1177_17470218231211573 for Poor maternal mental health is associated with a low degree of proactive control in refugee children by Gustaf Gredebäck, Marcus Lindskog and Jonathan Hall in Quarterly Journal of Experimental Psychology

sj-xlsx-2-qjp-10.1177_17470218231211573 – Supplemental material for Poor maternal mental health is associated with a low degree of proactive control in refugee children Supplemental material, sj-xlsx-2-qjp-10.1177_17470218231211573 for Poor maternal mental health is associated with a low degree of proactive control in refugee children by Gustaf Gredebäck, Marcus Lindskog and Jonathan Hall in Quarterly Journal of Experimental Psychology

## Notes

1. There are many alternative dependent variables that can be derived from the AX-CPT task, but in this article, we have selected the dependent variable that we believe more clearly index degree of proactive control (d′). Given the large number of statistical models required to answer the current research question, we have refrained from assessing (and thus from reporting) any of the other potential dependent variables from this task. However, error rates and response times (RTs) are reported on a descriptive level in Table 2.

2. The variable group indicates whether the participant is a child, a mother, or a father, within each family.

3. The label mother/father indicates that two separate variables were included in the model, one for mothers and another for fathers, and should be read as, for example, potentially traumatic events reported by mothers + potentially traumatic events reported by fathers + in the specification. The term OR specifies that the model was run with mothers or fathers in separate models.

4. An extended version of model C3 (suggested by reviewers) that control for children’s fluid intelligence (Table 1) illustrates an association between fluid intelligence and d′, *F*(1,130.3) = 9.95, *p* = .002, but this association did not impact or interact with the findings reported earlier.

## References

[bibr1-17470218231211573] Astor K. Lindskog M. Forssman L. Kenward B. Fransson M. Skalkidou A. Tharner A. Cassé J. Gredebäck G. (2020). Social and emotional contexts predict the development of gaze following in early infancy: A social-first account of gaze following. Royal Society Open Science, 7(9), 1–11. 10.1098/rsos.201178 PMC7540771 33047063

[bibr2-17470218231211573] Astor K. Lindskog M. Juvrud J. Wangchuk Namgyel S. C. Wangmo T. Tshering K. P. Gredebäck G. (2022). Maternal postpartum depression impacts infants’ joint attention differentially across cultures. Developmental Psychology, 58, 2230–2238. 10.1037/dev0001413 36107661

[bibr3-17470218231211573] Banerjee A. V. Duflo E. (2012). Poor economics. PublicAffairs.

[bibr4-17470218231211573] Berg L. Charboti S. Montgomery E. Hjern A. (2019). Parental PTSD and school performance in 16-year-olds—A Swedish national cohort study. Nordic Journal of Psychiatry, 73(4–5), 264–272. 10.1080/08039488.2019.1620852 31134834

[bibr5-17470218231211573] Bradley R. H. Corwyn R. F. (2002). Socioeconomic status and child development. Annual Review of Psychology, 53, 371–399. 10.1146/annurev.psych.53.100901.135233 11752490

[bibr6-17470218231211573] Braver T. S. (2012). The variable nature of cognitive control: A dual mechanisms framework. Trends in Cognitive Sciences, 16(2), 106–113. 10.1016/j.tics.2011.12.010 22245618 PMC3289517

[bibr7-17470218231211573] Cann A. Calhoun L. G. Tedeschi R. G. Taku K. Vishnevsky T. Triplett K. N. Danhauer S. C. (2010). A short form of the Posttraumatic Growth Inventory. Anxiety, Stress, and Coping, 23(2), 127–137. 10.1080/10615800903094273 19582640

[bibr8-17470218231211573] Chatham C. H. Frank M. J. Munakata Y. (2009). Pupillometric and behavioral markers of a developmental shift in the temporal dynamics of cognitive control. Proceedings of the National Academy of Sciences of the United States of America, 106(14), 5529–5533. 10.1073/pnas.0810002106 19321427 PMC2666994

[bibr9-17470218231211573] Chen S. H. Cohodes E. Bush N. R. Lieberman A. F. (2020). Child and caregiver executive function in trauma-exposed families: Relations with children’s behavioral and cognitive functioning. Journal of Experimental Child Psychology, 200, 104946. 10.1016/j.jecp.2020.104946 32791380

[bibr10-17470218231211573] El-Khani A. Ulph F. Peters S. Calam R. (2016). Syria: The challenges of parenting in refugee situations of immediate displacement. Intervention, 14(2), Article 2. 10.1097/WTF.0000000000000118

[bibr11-17470218231211573] Eltanamly H. Leijten P. Jak S. Overbeek G. (2021). Parenting in times of war: A meta-analysis and qualitative synthesis of war exposure, parenting, and child adjustment. Trauma, Violence, & Abuse, 22(1), 147–160. 10.1177/1524838019833001 PMC7675766 30852950

[bibr12-17470218231211573] Fales C. L. Barch D. M. Burgess G. C. Schaefer A. Mennin D. S. Gray J. R. Braver T. S. (2008). Anxiety and cognitive efficiency: Differential modulation of transient and sustained neural activity during a working memory task. Cognitive, Affective, & Behavioral Neuroscience, 8(3), 239–253. 10.3758/CABN.8.3.239 18814461

[bibr13-17470218231211573] Filippi C. A. Subar A. Ravi S. Haas S. Troller-Renfree S. V. Fox N. A. Leibenluft E. Pine D. S. (2022). Developmental changes in the association between cognitive control and anxiety. Child Psychiatry & Human Development, 8, 599–609. 10.1007/s10578-021-01150-5 PMC9107422 33738691

[bibr14-17470218231211573] Friedman N. P. Miyake A. Young S. E. DeFries J. C. Corley R. P. Hewitt J. K. (2008). Individual differences in executive functions are almost entirely genetic in origin. Journal of Experimental Psychology: General, 137(2), 201–225. 10.1037/0096-3445.137.2.201 18473654 PMC2762790

[bibr15-17470218231211573] Frounfelker R. L. Miconi D. Farrar J. Brooks M. A. Rousseau C. Betancourt T. S. (2020). Mental health of refugee children and youth: Epidemiology, interventions, and future directions. Annual Review of Public Health, 41(1), Article 1. 10.1146/annurev-publhealth-040119-094230 PMC9307067 31910713

[bibr16-17470218231211573] Garg A. Dworkin P. H. (2011). Applying surveillance and screening to family psychosocial issues: Implications for the medical home. Journal of Developmental and Behavioral Pediatrics: JDBP, 32(5), 418–426. 10.1097/DBP.0b013e3182196726 21522019 PMC3111883

[bibr17-17470218231211573] Gonthier C. Macnamara B. N. Chow M. Conway A. R. A. Braver T. S. (2016). Inducing proactive control shifts in the AX-CPT. Frontiers in Psychology, 7, Article 1822. 10.3389/fpsyg.2016.01822 PMC5118587 27920741

[bibr18-17470218231211573] Gonthier C. Zira M. Colé P. Blaye A. (2019). Evidencing the developmental shift from reactive to proactive control in early childhood and its relationship to working memory. Journal of Experimental Child Psychology, 177, 1–16. 10.1016/j.jecp.2018.07.001 30165288

[bibr19-17470218231211573] Gottwald J. M. Achermann S. Marciszko C. Lindskog M. Gredebäck G. (2016). An embodied account of early executive-function development. Psychol Sci, 27(12), 1600–1610. 10.1177/0956797616667447 27765900 PMC5154392

[bibr20-17470218231211573] Gredebäck G. Falck-Ytter T. (2015). Eye movements during action observation. Perspect Psychol Sci, 10(5), 591–598. 10.1177/1745691615589103 26385998 PMC4576502

[bibr21-17470218231211573] Gredebäck G. Haas S. Hall J. Pollak S. Karakus D. C. Lindskog M. (2021). Social cognition in refugee children: An experimental cross-sectional study of emotional processing with Syrian families in Turkish communities. Royal Society Open Science, 8(8), 210362. 10.1098/rsos.210362 34386252 PMC8334827

[bibr22-17470218231211573] Gredebäck G. Hall J. Lindskog M. (2022). Fluid intelligence in refugee children. A cross-sectional study of potential risk and resilience factors among Syrian refugee children and their parents. Intelligence, 94, 101684. 10.1016/j.intell.2022.101684

[bibr23-17470218231211573] Gugelberg H. M. von Schweizer K. Troche S. J. (2021). The dual mechanisms of cognitive control and their relation to reasoning and the item-position effect. Acta Psychologica, 221, 103448. 34784536 10.1016/j.actpsy.2021.103448

[bibr24-17470218231211573] Haas S. A. Amso D. Fox N. A. (2017). The effects of emotion priming on visual search in socially anxious adults. Cognition & Emotion, 31(5), 1041–1054. 10.1080/02699931.2016.1180281 27198991 PMC5116285

[bibr25-17470218231211573] Heckman J. Pinto R. Savelyev P. (2013). Understanding the mechanisms through which an influential early childhood program boosted adult outcomes. American Economic Review, 103(6), 2052–2086. 10.1257/aer.103.6.2052 24634518 PMC3951747

[bibr26-17470218231211573] Husa R. A. Buchanan T. W. Kirchhoff B. A. (2022). Subjective stress and proactive and reactive cognitive control strategies. European Journal of Neuroscience, 55(9–10), 2558–2570. 10.1111/ejn.15214 33783883

[bibr27-17470218231211573] Jacob T. Johnson S. L. (1997). Parent–child interaction among depressed fathers and mothers: Impact on child functioning. Journal of Family Psychology, 11, 391–409. 10.1037/0893-3200.11.4.391 11322084

[bibr28-17470218231211573] Karr J. E. Areshenkoff C. N. Rast P. Hofer S. M. Iverson G. L. Garcia-Barrera M. A. (2018). The unity and diversity of executive functions: A systematic review and re-analysis of latent variable studies. Psychological Bulletin, 144(11), 1147–1185. 10.1037/bul0000160 30080055 PMC6197939

[bibr29-17470218231211573] Kraay A. McKenzie D. (2014). Do poverty traps exist? Assessing the evidence. Journal of Economic Perspectives, 28(3), 127–148. 10.1257/jep.28.3.127

[bibr30-17470218231211573] Kubota M. Hadley L. V. Schaeffner S. Könen T. Meaney J.-A. Auyeung B. Morey C. C. Karbach J. Chevalier N. (2020). Consistent use of proactive control and relation with academic achievement in childhood. Cognition, 203, 104329. 10.1016/j.cognition.2020.104329 32526518

[bibr31-17470218231211573] Lang A. J. Stein M. B. (2005). An abbreviated PTSD checklist for use as a screening instrument in primary care. Behaviour Research and Therapy, 43(5), 585–594. 10.1016/j.brat.2004.04.005 15865914

[bibr32-17470218231211573] Leen-Feldner E. W. Feldner M. T. Knapp A. Bunaciu L. Blumenthal H. Amstadter A. B. (2013). Offspring psychological and biological correlates of parental posttraumatic stress: Review of the literature and research agenda. Clinical Psychology Review, 33(8), 1106–1133. 10.1016/j.cpr.2013.09.001 24100080

[bibr33-17470218231211573] Lieder F. Iwama G. (2021). Toward a formal theory of proactivity. Cognitive, Affective, & Behavioral Neuroscience, 21(3), 490–508. 10.3758/s13415-021-00884-y PMC8208939 33721229

[bibr34-17470218231211573] Lorsbach T. C. Reimer J. F. (2008). Context processing and cognitive control in children and young adults. The Journal of Genetic Psychology, 169(1), 34–50. 10.3200/GNTP.169.1.34-50 18476476

[bibr35-17470218231211573] Lorsbach T. C. Reimer J. F. (2010). Developmental differences in cognitive control: Goal representation and maintenance during a continuous performance task. Journal of Cognition and Development, 11(2), 185–216. 10.1080/15248371003699936

[bibr36-17470218231211573] Lucenet J. Blaye A. (2014). Age-related changes in the temporal dynamics of executive control: A study in 5- and 6-year-old children. Frontiers in Psychology, 5, Article 831. https://www.frontiersin.org/article/10.3389/fpsyg.2014.00831 10.3389/fpsyg.2014.00831 PMC4114259 25120523

[bibr37-17470218231211573] Lundborg P. Plug E. Rasmussen A. W. (2021). On the family origins of human capital formation: Evidence from donor children (SSRN Scholarly Paper ID 3921498). Social Science Research Network. https://papers.ssrn.com/abstract=3921498

[bibr38-17470218231211573] Matheny A. P. Wachs T. D. Ludwig J. L. Phillips K. (1995). Bringing order out of chaos: Psychometric characteristics of the confusion, hubbub, and order scale. Journal of Applied Developmental Psychology, 16(3), 429–444. 10.1016/0193-3973(95)90028-4

[bibr39-17470218231211573] Michalek J. Lisi M. Binetti N. Ozkaya S. Hadfield K. Dajani R. Mareschal I. (2022). War-related trauma linked to increased sustained attention to threat in children. Child Development, 93(4), 900–909. 10.1111/cdev.13739 35147214 PMC9542223

[bibr40-17470218231211573] Mollica R. F. Caspi-Yavin Y. Bollini P. Truong T. Tor S. Lavelle J. (1992). The Harvard Trauma Questionnaire: Validating a cross-cultural instrument for measuring torture, trauma, and posttraumatic stress disorder in Indochinese refugees. Journal of Nervous and Mental Disease, 180(2), 111–116. 10.1097/00005053-199202000-00008 1737972

[bibr41-17470218231211573] Mott F. L. (2004). The utility of the HOME-SF scale for child development research in a large national longitudinal survey: The National Longitudinal Survey of Youth 1979 cohort. Parenting, 4(2–3), 259–270. 10.1080/15295192.2004.9681273

[bibr42-17470218231211573] Mueller S. C. Unal C. Saretta M. Al Mughairbi F. Gómez-Odriozola J. Calvete E. Metin B. (2021). Working memory and emotional interpretation bias in a sample of Syrian refugee adolescents. European Child & Adolescent Psychiatry, 30(12), 1885–1894. 10.1007/s00787-020-01656-8 33025075

[bibr43-17470218231211573] Nelson C. A. Gabard-Durnam L. J. (2020). Early adversity and critical periods: Neurodevelopmental consequences of violating the expectable environment. Trends in Neurosciences, 43(3), Article 3. 10.1016/j.tins.2020.01.002 PMC8092448 32101708

[bibr44-17470218231211573] Ossmy O. Kaplan B. E. Han D. Xu M. Bianco C. Mukamel R. Adolph K. E. (2022). Real-time processes in the development of action planning. Current Biology, 32(1), 190–199.e3. 10.1016/j.cub.2021.11.018 34883048

[bibr45-17470218231211573] Parfitt Y. Pike A. Ayers S. (2014). Infant developmental outcomes: A family systems perspective. Infant and Child Development, 23(4), 353–373. 10.1002/icd.1830

[bibr46-17470218231211573] Peltonen K. Gredebäck G. Pollak S. D. Lindskog M. Hall J. (2023). The role of maternal trauma and discipline types in emotional processing among Syrian refugee children. European Child & Adolescent Psychiatry, 32, 1487–1495. 10.1007/s00787-022-01962-3 35217919 PMC10326120

[bibr47-17470218231211573] Qouta S. R. Vänskä M. Diab S. Y. Punamäki R.-L. (2021). War trauma and infant motor, cognitive, and socioemotional development: Maternal mental health and dyadic interaction as explanatory processes. Infant Behavior and Development, 63, 101532. 10.1016/j.infbeh.2021.101532 33588286

[bibr48-17470218231211573] Sack W. H. Clarke G. N. Seeley J. (1995). Posttraumatic stress disorder across two generations of Cambodian refugees. Journal of the American Academy of Child and Adolescent Psychiatry, 34(9), 1160–1166. 10.1097/00004583-199509000-00013 7559310

[bibr49-17470218231211573] Salo V. C. Schunck S. J. Humphreys K. L. (2020). Depressive symptoms in parents are associated with reduced empathy toward their young children. PLOS ONE, 15(3), Article e0230636. 10.1371/journal.pone.0230636 PMC7089529 32203542

[bibr50-17470218231211573] Save the Children. (2021). Stop the war on children: A crisis of recruitment. Save the Children’s Resource Centre. https://resourcecentre.savethechildren.net/document/stop-the-war-on-children-a-crisis-of-recruitment/

[bibr51-17470218231211573] Scharpf F. Mueller S. C. Hecker T. (2022). The executive functioning of Burundian refugee youth: Associations with individual, family and community factors. Journal of Applied Developmental Psychology, 80, 101399. 10.1016/j.appdev.2022.101399

[bibr52-17470218231211573] Shields G. S. Sazma M. A. Yonelinas A. P. (2016). The effects of acute stress on core executive functions: A meta-analysis and comparison with cortisol. Neuroscience and Biobehavioral Reviews, 68, 651–668. 10.1016/j.neubiorev.2016.06.038 27371161 PMC5003767

[bibr53-17470218231211573] Siebert J. U. Kunz R. E. Rolf P. (2020). Effects of proactive decision making on life satisfaction. European Journal of Operational Research, 280(3), 1171–1187. 10.1016/j.ejor.2019.08.011

[bibr54-17470218231211573] Slone M. Mann S. (2016). Effects of war, terrorism and armed conflict on young children: A systematic review. Child Psychiatry and Human Development, 47(6), 950–965. 10.1007/s10578-016-0626-7 26781095

[bibr55-17470218231211573] Sosic-Vasic Z. Kröner J. Schneider S. Vasic N. Spitzer M. Streb J. (2017). The Association between parenting behavior and executive functioning in children and young adolescents. Frontiers in Psychology, 8, Article 472. https://www.frontiersin.org/article/10.3389/fpsyg.2017.00472 10.3389/fpsyg.2017.00472 PMC5371664 28424644

[bibr56-17470218231211573] Tornau K. Frese M. (2013). Construct clean-up in proactivity research: A meta-analysis on the nomological net of work-related proactivity concepts and their incremental validities. Applied Psychology, 62(1), 44–96. 10.1111/j.1464-0597.2012.00514.x

[bibr57-17470218231211573] Troller-Renfree S. V. Buzzell G. A. Fox N. A. (2020). Changes in working memory influence the transition from reactive to proactive cognitive control during childhood. Developmental Science, 23(6), Article e12959. 10.1111/desc.12959 32141641

[bibr58-17470218231211573] Tu H.-F. Skalkidou A. Lindskog M. Gredebäck G. (2021). Maternal childhood trauma and perinatal distress are related to infants’ focused attention from 6 to 18 months. Scientific Reports, 11(1), 24190. 10.1038/s41598-021-03568-2 34921204 PMC8683435

[bibr59-17470218231211573] United Nations International Children’s Emergency Fund. (2022). Child displacement and refugees. https://data.unicef.org/topic/child-migration-and-displacement/displacement/

[bibr60-17470218231211573] van der Waerden J. Bernard J. Y. De Agostini M. Saurel-Cubizolles M.-J. Peyre H. Heude B. Melchior M. , & EDEN Mother-Child Cohort Study Group. (2017). Persistent maternal depressive symptoms trajectories influence children’s IQ: The EDEN mother-child cohort. Depression and Anxiety, 34(2), 105–117. 10.1002/da.22552 27603172

[bibr61-17470218231211573] van Ee E. Kleber R. J. Jongmans M. J. Mooren T. T. M. Out D. (2016). Parental PTSD, adverse parenting and child attachment in a refugee sample. Attachment and Human Development, 18(3), 273–291. 10.1080/14616734.2016.1148748 26982876

[bibr62-17470218231211573] van Ee E. Kleber R. J. Mooren T. T. (2012). War trauma lingers on: Associations between maternal posttraumatic stress disorder, parent-child interaction, and child development. Infant Mental Health Journal, 33(5), 459–468. 10.1002/imhj.21324 28520264

[bibr63-17470218231211573] van Ravenzwaaij D. Dutilh G. Wagenmakers E.-J . (2011). Cognitive model decomposition of the BART: Assessment and application. Journal of Mathematical Psychology, 55(1), 94–105. 10.1016/j.jmp.2010.08.010

[bibr64-17470218231211573] von Hofsten C . (2004). An action perspective on motor development. Trends in Cognitive Sciences, 8(6), 266–272. 10.1016/j.tics.2004.04.002 15165552

[bibr65-17470218231211573] Wechsler D. (1999). Wechsler abbreviated scale of intelligence. The Psychological Corporation.

[bibr66-17470218231211573] Woods D. L. Wyma J. M. Yund E. W. Herron T. J. Reed B. (2015). Factors influencing the latency of simple reaction time. Frontiers in Human Neuroscience, 9, Article 131. https://www.frontiersin.org/articles/10.3389/fnhum.2015.00131 10.3389/fnhum.2015.00131 PMC4374455 25859198

[bibr67-17470218231211573] Yang Y. Miskovich T. A. Larson C. L. (2018). State anxiety impairs proactive but enhances reactive control. Frontiers in Psychology, 9, Article 2570. 10.3389/fpsyg.2018.02570 PMC6300490 30618987

[bibr68-17470218231211573] Yaylaci F. T. (2018). Trauma and resilient functioning among Syrian refugee children. Development and Psychopathology, 30(5), Article 5. 10.1017/S0954579418001293 30295224

[bibr69-17470218231211573] Young E. S. Frankenhuis W. E. DelPriore D. J. Ellis B. J. (2022). Hidden talents in context: Cognitive performance with abstract versus ecological stimuli among adversity-exposed youth. Child Development, 93(5), 1493–1510. 10.1111/cdev.13766 35404500 PMC9543758

