# Supplementary material for: Poor maternal mental health is associated with a low degree of proactive control in refugee children
Source: Q J Exp Psychol (Hove). 2023 Nov 29;77(10):1987–99. doi: 10.1177/17470218231211573 (PMC11462783; doi:10.1177/17470218231211573)
Supplement: sj-docx-1-qjp-10.1177_17470218231211573 – Supplemental material for Poor maternal mental health is associated with a low degree of proactive control in refugee children [file sj-docx-1-qjp-10.1177_17470218231211573.docx]

Supplementary Material for:

**Poor maternal mental health is associated with a low degree of proactive control in refugee children.**

Gustaf Gredebäck, Marcus Lindskog, and Jonathan Hall

Uppsala Universitet, Sweden

**Corresponding Author:**

Gustaf Gredebäck

Uppsala Universitet

Uppsala 751 05

Sweden

<https://orcid.org/0000-0003-3046-0043>

gustaf.gredeback@psyk.uu.se

Extraction of AX-CPT-data

Summary of most important variables:

- **AX_errorrate** = error rate 0-1 in AX trials
- **AX_average_RTprobe** = reaction time in valid AX trials (ms)
- **AY_errorrate** = error rate 0-1 in AY trials
- **AY_average_RTprobe** = reaction time in valid AY trials (ms)
- **BX_errorrate** = error rate 0-1 in BX trials
- **BX_average_RTprobe** = reaction time in valid BX trials (ms)
- **BY_errorrate** = error rate 0-1 in BY trials
- **BY_average_RTprobe** = reaction time in valid BY trials (ms)
- **d_context** = as described below
- **A_cue_bias** = as described below
- **proactive_behavioral_index_RT** = as described below, (PBI reaction time in article)
- **proactive_behavioral_index_error_rate** = as described below (PBI error rate in article)

Date: 2021-10-22

All data was stored in a database at Uppsala University on a long format. I.e. all trials were stored as separate rows with columns including all necessary data such as subject id, parent id, trial type (AX/BX/AY/BY), response times for cues/probes, and response types for cues/probes (correct/incorrect). Practice trials (15 per subject) were removed before analysis, and trials with response times <250ms were excluded. The threshold of 250ms was based on visual inspection of response time histograms which showed a rapid increase in frequency for response times >300ms.

For the infant data (n=213) 388 of 12900 trials (3.0%) were excluded due to <250ms RTs. The resulting dataset consisted of 12512 trials for the infant data, with the following nr trials in the different conditions:

Nr AX_long: 2511

Nr AX_short: 2494

Nr AY_long: 624

Nr AY_short: 619

Nr BX_long: 629

Nr BX_short: 623

Nr BY_long: 2521

Nr BY_short: 2491

For the parent data (n=148) 168 of 8880 trials (1.9%) were excluded due to <250ms RTs. The resulting dataset consisted of 8712 trials for the parent data, with the following nr trials in the different conditions:

Nr AX_long: 1756

Nr AX_short: 1725

Nr AY_long: 437

Nr AY_short: 428

Nr BX_long: 442

Nr BX_short: 430

Nr BY_long: 1760

Nr BY_short: 1734

The algorithm had a parameter switch to include trials with long delays (5500ms) or short delays (1500ms) or all trials (both 5500ms and 1500ms). The analysis generated two tables for further analysis in other statistical software:

1. a table with all trials on a long format (for analysis using LMMs etc). This table is a data dump from the database, but with correct/incorrect classifications and cleaned variable names.
2. a table with one participant per row with averaged data and summary measures (for analysis using ANOVAs etc). The summary measures are created as described below.

The extraction of measures were done according to the article “Inducing Proactive Control Shifts in the AX-CPT”, <https://www.frontiersin.org/articles/10.3389/fpsyg.2016.01822/full> . Analysis pipeline was double checked by the first author of this article. The analysis code is presented below, together with explanatory quotes from the article in green.

% In order to correct for trials where error

% rates were equal to zero, a log-linear correction was applied to all error rate data prior

% to computing the d′-context, the A-cue bias and the PBIs (as in Braver et al., 2009; see also Hautus, 1995).

% This correction was applied as error rate = (number of errors + 0.5)/(number of trials + 1).

errorrateAX = (nrAXincorrect +0.5)/(nrAXtrials+1);

errorrateBX = (nrBXincorrect +0.5)/(nrBXtrials+1);

errorrateAY = (nrAYincorrect +0.5)/(nrAYtrials+1);

errorrateBY = (nrBYincorrect +0.5)/(nrBYtrials+1);

% The d′-context index was calculated by computing a d′ index from hits on AX trials

% and false alarms on BX trials as Z(H) - Z(F), with H representing hits on AX trials,

% F representing false alarms on BX trials, and Z representing the z-transform of a value.

% This measure reflects the ability of the participants to use contextual information

% from the cue to drive their answer on the probe (e.g., Barch et al., 2001)

%

% NOTE: The Matlab command for Z(H) is norminv(H).

indi(i).d_context = norminv(1-errorrateAX) - norminv(errorrateBX);

% An A-cue bias measure was also calculated (Richmond et al., 2015) by computing a

% c criterion from hits on AX trials and false alarms on AY trials as 1/2*(Z[H] + Z[F]),

% with H representing hits on AX trials and F representing false alarms on AY trials2.

% This measure reflects the tendency of participants to make a target response following an A

% cue, independently of the identity of the probe.

indi(i).A_cue_bias = 1/2*(norminv(1-errorrateAX) + norminv(errorrateAY));

% The third index was the PBI (Braver et al., 2009), calculated as (AY - BX)/(AY + BX).

% This index reflects the relative balance of interference between AY and BX trials:

% a positive PBI reflects higher interference on AY trials, indicating proactive control,

% whereas a negative PBI reflects higher interference on BX trials, indicating reactive

% control. The PBI was computed separately for error rates (based on average error rates on AY

% and BX trials)and for RTs (based on average RTs on AY and BX trials)

AY = indi(i).AY_average_RTprobe; % reaction time AY trials

BX = indi(i).BX_average_RTprobe; % reaction time BX trials

indi(i).proactive_behavioral_index_RT = (AY-BX)/(AY+BX);

AY = errorrateAY;

BX = errorrateBX;

indi(i).proactive_behavioral_index_error_rate = (AY-BX)/(AY+BX);

end
